# AI-assisted versus traditional radiographic interpretation for early caries detection

**DOI:** 10.6026/973206300222278

**Published:** 2026-04-30

**Authors:** Vikram Karande, Mohd Zeeshan Ahmad, Vishal Kulkarni, Priyanka Chandra, Sowjanya Gunukula, Mousami Kundu, Ritik Kashwani

**Affiliations:** 1 Department of Dentistry, AIIMS, Rajkot, Gujarat, India; 2 Department of Conservative Dentistry & Endodontics, Rama Dental College, Hospital & Research Centre, Kanpur, Uttar Pradesh, India; 3 Department of Oral and Maxillofacial Surgery, Army Dental Centre Research and Referral, Delhi Cantt, India; 4 Department of Conservative Dentistry and Endodontics, Kothiwal Dental college and Research Centre Kanth Road, Moradabad, Uttar Pradesh, India; 5 Private Partition, Flomo Dental Private Clinic 295 Ukiah St, Lewisville, Texas, USA; 6 Department of Oral & Maxillofacial Surgery, Awadh Dental College and Hospital, NH-33, Dalma, Bhilaipahari, Dalma, Jamshedpur, Jharkhand, India; 7 Department of Oral Medicine and Radiology, School of Dental Sciences, Sharda University, Greater Noida, Uttar Pradesh, India

**Keywords:** Artificial intelligence (AI), dental caries, diagnostic accuracy, early detection, radiographic interpretation

## Abstract

Early detection of dental caries is critical for implementing effective preventive measures, yet traditional radiographic interpretation often lacks consistency due to observer variability. Therefore, it is of interest to compare the diagnostic performance of AI-assisted and conventional radiographic interpretations for detecting early carious lesions. Hence, a total of 100 subjects were randomly assigned to either the AI-assisted or conventional interpretation group, with diagnostic accuracy assessed using sensitivity, specificity and overall accuracy. Thus, we show that AI-assisted interpretation outperformed conventional methods, with higher sensitivity (84%) and diagnostic accuracy (82%). This advancement highlights AI's potential to significantly improve early caries detection in dental practice.

## Background:

Caries affects people of all ages and walks of life worldwide. In fact, it is one of the most common chronic diseases. With the advancement in preventive dentistry, early carious lesions tend to go unnoticed until they reach more advanced stages requiring costly and invasive treatment [1]. Thus, detection of caries is essential because it may be effectively preventively managed when they are still small and have invaded only the enamel or dentine [2]. Taking X-ray pictures of lower and upper posterior teeth, i.e. bitewing radiography remains an important aid in the diagnosis of proximal and occlusal caries which is not clinically visible during an examination [3]. The interpretation of a conventional radiograph depends on the visual perception of a clinician. Even though experienced dentists are able to fairly accurately identify carious lesions, radiographic interpretation, through reliable, is subjective and subject to both inter-observer and intra-observer variability [4]. The performance of diagnostic imaging can be altered with shift in perspective due to image quality as well as artefacts [5]. The difficulties are particularly pronounced regarding the detection of early enamel and incipient dentinal caries with radiolucent changes being scanty and easily missed [6]. As a result, lesions at an early stage are often underdiagnosed or misdiagnosed, which results in a delay in treatment or unnecessary surgical intervention [7]. Artificial intelligence (AI), particularly the use of machine learning and deep learning algorithms, has attracted attention in recent years. The possibilities it promises for medical and dental diagnostics are discussed here [8]. AI-enabled radiographic interpretation systems are built to analyze massive imaging data, expect complex patterns and consistently highlight possible pathology areas [9]. The use of AI in dentistry has expanded rapidly to detect dental caries, periodontal bone loss, periapical lesions and oral diseases [10]. Designed to help clinicians with a diagnosis, these systems are not put in place to replace that judgement [11]. Many early and observational studies have shown that these AI-assisted systems may have easier detection of early carious lesions than visual interpretation alone [12].

AI tools may be able to detect nuanced radiographic changes that the human eye may not see which lowers the chances of missed diagnoses [13]. Additionally, AI-supported interpretation has benefits of standardization, decreased observer variability, enhanced efficiency and other aspects which can be beneficial for clinical trials with heavy volumes and community-based screening programs [14]. For novice dental students and practitioners alike, AI support may serve as a hands-on learning tool and provide assistance in reducing the gap in diagnosis √between beginner and expert clinician [15]. The reliability, generalizability and clinical utility of AI-assisted radiographic interpretation remain a concern notwithstanding these potential benefits. Numerous studies already executed rely heavily on laboratory data and metadata image datasets, which do not replicate real-world clinical settings [16]. The accuracy of a diagnosis can be affected due to radiographic techniques, anatomy, caries and image quality [17]. In addition, if AI systems are integrated into clinical workflows without caution you may get false positives, overtreatment and impaired clinical reasoning. Essentially, there must be strong clinical evidence to objectively assess whether there is any improvement with AI- assisted interpretation over standard interpretation [18]. According to the gold standard methodology for assessing diagnostic interventions, randomized controlled trials (RCTs) minimize bias and directly compare competing diagnostic interventions under ideal conditions [19]. Regarding the early detection of caries, an RCT comparing AI-assisted and conventional radiographic interpretation could provide high-quality evidence on differences in diagnostic accuracy, sensitivity, specificity and other overall clinical utility. The purpose of these trials is to understand whether AI's theoretical benefits should entail practical benefits for patients [20]. Moreover, it is essential to take into account the ethical, educational and medico-legal issues. It must be clarified how much influence AI should have on the clinical judgments of a dentist, while the dentist still has final responsibility for diagnosis or treatment decisions [21]. Studies with credible evidence by way of RCTs can inform clinical guidelines, regulatory frameworks and educational curricular, contributing towards the safe and effective adoption of AI [22]. The problems of dental caries worldwide, limitations of conventional radiographic interpretation and rapid development of an AI-based diagnostic tool, there is a strong need to evaluate the role of AI in early detection of caries using rigorous clinical trials [23]. Therefore, it is of interest to report the comparative diagnostic accuracy and clinical effectiveness of AI-assisted radiographic interpretation and conventional radiographic interpretation early detection of dental caries.

## Methodology:

This study has been designed as a single-center; parallel group randomized controlled trial to compare radiographic AI-assisted interpretation versus conventional radiographic interpretation in detecting early dental caries. The study was conducted in the Department of Oral Medicine and Radiology of a dental teaching institution after obtaining approval from the Institutional Ethics Committee. The Declaration of Helsinki guided how the trial was made and written informed consent was obtained from all trial participants. The total number of participants that were included in the study is 100. The sample size was selected for feasibility. Moreover, sample size was based on previous similar diagnostic accuracy studies for early caries detection using AI versus conventional interpretation. Participants underwent a consecutive sampling technique for recruiting the patients attending outpatient department for a routine dental examination. The inclusion criteria of our study were adults aged 18-45 years with at least two posterior teeth suitable for bitewing radiographic examination and no history of restorative treatment on the selected tooth surfaces. We excluded patients with large carious lesions. We also excluded patients with extensive restorations such as crowns or orthodontic appliances that interfere with the visibility of the radiograph. Patients with developmental enamel defects and a systemic condition that interfered with the mineralization of the tooth were also excluded. Lastly, we did not take patients with oblique, poor-quality or distorted radiographs. Using a computer-generated randomization sequence, 100 participants were randomly allocated into 2 equal groups (n = 50 each) Orthopantomogram interpretation in Group A was aided by artificial intelligence, while Group B underwent conventional interpretation. The researchers concealed allocation through the use of sealed opaque envelopes which were opened during the analysis. For every participant, standardized digital bitewing radiographs were obtained using the same X-ray unit, exposure factors and positioning technique to minimize variability. All radiographs were taken by a radiographic technician and checked for adequate quality for inclusion in the study. In Group A, dental radiographs were assessed using well-validated AI-based software for early detection of caries. The software was able to automatically highlight the carious lesions suspected by the dentist which were confirmed by a calibrated examiner with reference to AI output. In Group B, the same examiner interpreted the radiographs in a conventional manner without the help of an AI system, relying only on the human eye and clinical experience. To ensure uniformity, all radiographs were used for interpretation prior to the study calibration by a separate examiner. The same radiographs were interpreted under different methods with a washout period of two weeks to reduce recall bias. The group allocation was blind during evaluation of the examiner. Visual-tactile clinical examination of the tooth along with fiber-optic transillumination (when deemed necessary) served as the gold standard for diagnosis of early carious lesions. Lesions were classified using standard caries diagnostic criteria. The main outcome measure in this study will be diagnostic accuracy of early detection of caries, expressed in sensitivity specificity positive predictive value and negative predictive value. Other outcomes were agreement between methods and the time of reading. The information was entered in a spreadsheet and analysed statistically. The summary of the demographic variables made use of descriptive statistics. Both group diagnostic accuracy parameters were calculated and compared using appropriate statistical tests and p < 0.05 was considered statistically significant. According to the methodology devised, reliable evidence will be produced on the effectiveness of AI-assisted radiographic interpretation in early caries detection compared to conventional radiographic interpretation.

## Results:

The final analysis included a total of 100 participants with 50 radiographs interpreted with AI-assisted radiographic interpretation (Group A) and 50 interpreted with conventional radiographic interpretation (Group B). Each radiograph was of acceptable diagnostic quality and was used for statistical analysis. The demographic distribution of the study participants is summarized in (Table 1 - see PDF). The mean age of participants in Group A was 29.6 ± 6.2 years, while in Group B it was 30.1 ± 5.8 years. Gender distribution was comparable between the two groups and no statistically significant difference was observed, indicating successful randomization. The distribution of early carious lesions detected by both methods is presented. AI-assisted interpretation detected a higher number of early enamel and dentinal lesions compared to conventional interpretation. The difference was statistically significant (p < 0.05). As shown in (Table 2 - see PDF), AI-assisted interpretation demonstrated superior performance in identifying early carious changes when compared to the conventional method. Diagnostic accuracy parameters of both methods, calculated against the reference standard. AI-assisted interpretation demonstrated higher sensitivity (84%) compared to conventional interpretation (62%), while specificity was comparable between the two groups. As indicated in (Table 3 - see PDF), AI-assisted radiographic interpretation achieved higher overall diagnostic accuracy than the conventional approach. A binary logistic regression analysis was performed using STATA to evaluate the association between interpretation method and correct detection of early caries. The findings are summarized in (Table 4 - see PDF). AI-assisted interpretation showed significantly higher odds of correctly detecting early carious lesions compared to conventional interpretation. The mean time required for radiographic interpretation was significantly lower in the AI-assisted group. The STATA analysis demonstrated that radiographs interpreted with AI assistance were approximately 2.8 times more likely to result in correct early caries detection compared to conventional interpretation (Table 4 - see PDF). As evident from (Table 5 - see PDF), AI-assisted interpretation significantly reduced the time required for diagnosis compared to the conventional method. Overall, the results indicate that AI-assisted radiographic interpretation significantly improves the detection rate, diagnostic accuracy and efficiency of early caries diagnosis when compared with conventional radiographic interpretation.

## Discussion:

The results of this randomized controlled trial demonstrate that AI-assisted radiographic interpretation significantly enhances the diagnostic performance and efficiency of early dental caries detection compared to conventional interpretation. In the present study, the AI-assisted group showed higher sensitivity, overall accuracy and reduced interpretation time. These findings align with a growing body of literature evaluating the role of artificial intelligence in dental caries diagnostics; yet also reveal variations in performance across different clinical and research settings. In comparison to Patel *et al.* (2025), [24] who reported superior sensitivity and improved diagnostic efficiency for AI-assisted interpretation of bitewing radiographs, the current study similarly observed enhanced detection of early carious lesions with AI support. Patel and colleagues analyzed 400 bitewing radiographs and found that AI achieved higher sensitivity (91.2% versus 84.6%) and markedly reduced interpretation time compared with human clinicians, although specificity was slightly greater in conventional readings. These trends reinforce the potential for AI tools to complement clinician judgment in clinical practice. Moreover, Zhang *et al.* (2024) [25] conducted a large prospective clinical evaluation using intraoral images from 4,361 teeth in 191 patients. Their AI model, based on MobileNet-v3 and U-net architectures, demonstrated high overall accuracy (93.4%) and strong specificity (95.65%), although sensitivity varied depending on tooth position and caries type. The current trial's findings of enhanced overall accuracy with AI are consistent with Zhang *et al.*'s results, although differences in imaging modality (radiographs versus intraoral images) and lesion types suggest that performance may vary across diagnostic contexts. In addition, Karakus *et al.* (2024) [26] utilized a deep learning-based YOLOv8 algorithm for automated detection of interproximal, occlusal and secondary caries on bitewing radiographs. Their system achieved high average precision, sensitivity and F1 scores (0.977, 0.932 and 0.954, respectively), and indicating that advanced AI frameworks can deliver reliable detection metrics comparable to those seen in the present study. While the current RCT focused on early caries rather than multiple lesion types, the similar high performance of deep learning models supports the broader utility of AI in radiographic caries detection. However, not all studies have shown unequivocal superiority of AI over human interpretation. Rodrigues *et al.* (2025) [27] compared human operators, AI algorithms and near-infrared imaging for interproximal caries detection using histological validation. In contrast to many positive AI studies, this research found that operator radiographic assessment and NIRI imaging outperformed the AI program in sensitivity and AUC metrics for certain lesion types. This discrepancy underscores that AI models may be sensitive to dataset composition and algorithm design and that current AI solutions do not consistently outperform human examiners in all diagnostic contexts. The systematic review and meta-analysis by Ammar and Kühnisch (2024) [28] synthesized data from 14 PubMed-indexed studies of AI-aided caries diagnosis on bitewing radiographs. The pooled sensitivity (0.87) and specificity (0.89) reported in that review are comparable to the sensitivity and specificity observed in the present study, reinforcing that AI models generally perform well in caries detection with clinical relevance. However, the review also emphasized substantial heterogeneity and risk of bias in existing studies, highlighting the need for standardized clinical trials like the current one to validate AI performance. In totality, these studies show that diagnosis of caries through AI-assisted interpretation can significantly boost the diagnostic performance for early detection, particularly in sensitivity and efficiency as was the case in the current RCT. Nonetheless, the differences in performance observed in some studies shows that AI tools should be continuously validated in different clinical situations and imaging settings. The usability of an AI model in a clinical scenario is determined not only by its performance but also its combination with the clinician's expertise and workflow. The results of our study are consistent with most current studies showing AI helps obtain a correct diagnosis faster and more accurately. Nevertheless, the performance of AI depends on their methodology and lesion type; our novel approach is evidence of this. This shows that AI has the potential to supplement standard radiographic interpretation, especially for early detection of caries. This also suggests some work that should be undertaken as part of future research to fine-tune algorithms.

## Conclusion:

AI-assisted radiographic interpretation outperforms conventional methods in detecting early carious lesions with higher sensitivity and diagnostic accuracy. Specificity remained similar between both groups, while AI reduced interpretation time significantly. Routine use of AI can support clinicians in better diagnosing caries and improving clinical outcomes.
